# Current Limitations of Staph Infection Diagnostics, and the Role for VOCs in Achieving Culture-Independent Detection

**DOI:** 10.3390/pathogens12020181

**Published:** 2023-01-24

**Authors:** Carrie L. Jenkins, Heather D. Bean

**Affiliations:** 1 School of Life Sciences, Arizona State University, Tempe, AZ 85287, USA; 2 Center for Evolution and Medicine, Arizona State University, Tempe, AZ 85287, USA; 3 Center for Fundamental and Applied Microbiomics, The Biodesign Institute, Tempe, AZ 85287, USA

**Keywords:** *Staphylococcus aureus*, *Staphylococcus epidermidis*, coagulase negative staphylococci, diagnosis, volatile organic compounds, breath-based diagnostics, biomarkers, mVOC, culture-independent identification

## Abstract

Staphylococci are broadly adaptable and their ability to grow in unique environments has been widely established, but the most common and clinically relevant staphylococcal niche is the skin and mucous membranes of mammals and birds. *S. aureus* causes severe infections in mammalian tissues and organs, with high morbidities, mortalities, and treatment costs. *S. epidermidis* is an important human commensal but is also capable of deadly infections. Gold-standard diagnostic methods for staph infections currently rely upon retrieval and characterization of the infectious agent through various culture-based methods. Yet, obtaining a viable bacterial sample for *in vitro* identification of infection etiology remains a significant barrier in clinical diagnostics. The development of volatile organic compound (VOC) profiles for the detection and identification of pathogens is an area of intensive research, with significant efforts toward establishing breath tests for infections. This review describes the limitations of existing infection diagnostics, reviews the principles and advantages of VOC-based diagnostics, summarizes the analytical tools for VOC discovery and clinical detection, and highlights examples of how VOC biomarkers have been applied to diagnosing human and animal staph infections.

## 1. The Impacts of Staph on Humans and Non-Human Animals

Of the 62 or more species and 30 subspecies of staphylococci identified to date [1,2,3,4], the most prevalent, clinically relevant, and economically impactful staphylococcal species are *Staphylococcus aureus* and *S. epidermidis,* whose principal niches are the skin and mucous membranes of mammals and birds [1,5,6,7]. *S. aureus* and *S. epidermidis* commonly reside asymptomatically upon humans and animals, conferring no ill effects [8,9,10]. *S. aureus* is the predominant staph species isolated from the nares of adults and more frequently also from the nares of children [11]. *S. epidermidis* comprises nearly 75% of staph isolated from human head and axillae, up to 45% of the staph found on the legs and arms, and nearly 100% of the staph detected in the nares when *S. aureus* is absent [11]. Yet, these microbes are also colonizing opportunistic pathogens lying in wait until their host’s epithelia, immune system or microbiota fail to maintain homeostasis [10,12,13]. Upon breaching the epithelial or epidermal barriers, *S. aureus* can invasively infect nearly every mammalian tissue or organ with high morbidities, mortalities, and treatment costs [14]. Persons with chronic conditions, such as HIV, intravenous drug users, those with diabetes mellitus type I, and hemodialysis patients, experience a substantially increased risk of *S. aureus* infections due to compromised immunity, frequent skin and soft tissue breaches and infections, and greater colonization rates [15,16,17,18]. *S. aureus* can also establish chronic infections in persons with structural lung disease, such as cystic fibrosis (CF), non-CF bronchiectasis, and chronic obstructive pulmonary disease (COPD) [19,20,21]. Approximately 70% of CF patients produced a positive bacterial culture for methicillin-sensitive or resistant *S. aureus* lung infection during 2019 [22].

*S. aureus* also causes severe diseases in animals such as arthritis, toxic shock syndrome, urinary tract infections, and omphalitis, an infection of the umbilicus or surrounding tissues [23]. *S. aureus* is also the predominant infectious pathogen causing clinical and subclinical intramammary mastitis in dairy cattle globally [23,24,25,26,27,28,29,30,31,32,33], resulting in sizable economic losses. Liebe and colleagues estimate that the US economy loses $2 billion annually due to bovine mastitis, with worldwide losses of approximately $34 billion (USD) [34]. Further, these figures are estimated based on a disease incidence range of 25 to 41 cases per 100 cows per lactation, which ignores the myriad cases of subclinical mastitis with no apparent symptomology, but slight decline in milk production and increase in somatic cell count (a quantitative measure of milk quality based on presence of host (immune) cells) [35]. The economic losses due to subclinical cases are even greater than losses due to clinical cases [36]. 

Haag recently reported that *S. aureus* carriage rates in animals vary by host species. It is extremely prevalent, with nearly 90% carriage in chickens, 42% in pigs, 29% in sheep, and 23% in cows and heifers [37]. Animal carriers provide reservoirs for *S. aureus* mutation and adaptation resulting in new virulence traits and antimicrobial resistance in zoonotic infections [37], with *S. aureus* host-switching events between humans and domesticated animals causing significant concern [38,39,40,41]. For example, Akkou and colleagues recently demonstrated a new host shift of *S. aureus* causing bovine mastitis acquired from the animals’ caretakers [42], with 45.9% of the infections in cows and 29.8% of mastitic milk specimens being subclinical [42]. Furthermore, DNA micro-array analysis identified the human epidemic associated Clonal Complex 22 (CC22) present in both human (18% nasal carriage rate) and bovine isolates, indicating human transfer to dairy cattle [42]. In a second example of human-to-animal transmission, Viana and colleagues identified a naturally occurring single nonsynonymous nucleotide mutation within the human *S. aureus* clonal complex CC121 (*dltB* gene) [43], which converted the strain from non-infectious to epidemic-level virulence in farmed rabbits. 

The coagulase negative staph (CoNS) are ubiquitous commensals and have been commonly regarded as clinical sample contaminants when testing for bacterial infections [1,5,44,45,46]. However, relatively recently (post-1960s), select species of CoNS have been assigned pathogenic status (*S. epidermidis, S. saprophyticus, S. lugdunensis* and *S. schleiferi*), resulting from enhanced recognition as etiological agents of infections associated with medical interventions, such as implanted medical devices and immunosuppression [1,5,7,45]. Specifically, *S. epidermidis* is regarded to have intermediary virulence, falling between true apathogenic species, like *S. carnosus*, and highly virulent pathogenic species, such as *S. aureus* [47,48]. *S. epidermidis* is the predominant cause of hospital-associated infections, especially those with implanted medical devices as the primary reason for treatment [46,49]. Interestingly, clinical indications of *S. epidermidis* biofilm infections are distinct from *S. aureus*, with slight and lingering symptoms that lead to chronic, though indolent infection [46]. *S. epidermidis* may not produce as many toxins as *S. aureus,* and it may not demonstrate aggressive pathogenic characteristics like *S. aureus*; however, given identical infectious conditions, it can cause bacteremia leading to infectious endocarditis or sepsis [49,50].

## 2. Standard Diagnostic Methods for Staph Infections

### 2.1. Culture-Based Diagnostics

Vaccine development specific to *S. aureus* has been unsuccessful thus far, and therefore diagnosis and treatment of infections is the primary strategy for reducing morbidity and mortality caused by staph infections [51,52]. Canonical diagnostic approaches in infectious disease have centered upon retrieval and characterization of the infectious agent through various culture-based methods [53,54,55,56]. Once a specimen is obtained, identification of the infectious agent often begins with an enrichment culture in high-nutrient media to encourage amplification of the bacterial sample. Enrichment cultures require incubation at 37 °C for 18–24 h for fast-growing organisms, followed by secondary cultures and assays (e.g., biochemical testing for identification and antibiotic resistance testing) requiring an additional 12–48 h for identification. Selective and differential media, such as mannitol salt agar (MSA) for enrichment of *Staphylococcus* spp. and CHROMagar^TM^ Staph aureus for the direct differentiation and isolation of *S. aureus* from wound, tegument, and soft tissue specimens are essential tools for culture-based diagnostic procedures [57,58]. Although not intended for direct diagnosis, CHROMagar^TM^ Staph aureus aids in the differentiation and isolation of *S. aureus* from other bacteria – including other staph – from problematic samples that contain a multiplicity of bacterial pathogens, such as sputum from CF lung infections [57,58]. The enrichment of a single pink to mauve colony from a CHROMagar^TM^ plate or a mannitol-fermenting colony from MSA enables biochemical assays for diagnostic identification. The most common methods for identification of bacterial and yeast infections is the usage of API test panels, comprised of a series of individual miniaturized biochemical tests. The API Staph panel is inoculated from a single specimen colony grown from an enrichment culture plated on Columbia blood agar for 18 to 24 h, then suspended in API Staph Medium^®^ and incubated for an additional 18–24 h for the positive identification of 20 *Staphylococcus* species [59].

### 2.2. Molecular Detection

Genomic and proteomic methods are becoming more prevalent in clinical labs for the identification of staph infections and their clinically relevant traits, such as methicillin resistance. The GeneXpert^®^ MRSA/SA tests (Cepheid, Sunnyvale, CA, USA) for the diagnosis of blood or soft skin and tissue infections (SSTI) or nasal colonization are relatively recent entries into the diagnostic toolkit for *S. aureus* and methicillin-resistant *S. aureus* (MRSA) [54,60,61,62]. Coppens and colleagues demonstrated 93% sensitivity for *S. aureus* in about one hour from direct analysis of endotracheal aspirates, but suggested prior enrichment culturing to improve this value [61]. Bouza and colleagues utilized a rapid diagnostic algorithm of Gram stain and real-time PCR using the GeneXpert^®^ MRSA/SA SSTI molecular test, yielding results and antibiotic stewardship advice to clinicians with a median turnaround time of 4 h, compared to 99 h for enrichment culture diagnostics. The faster diagnosis resulted in shorter treatment regimens, lower antibiotic costs, lower length of hospital stay, and a 75.5% relative reduction in mortality [60]. 

Identification of the subspecies or strain level for staphylococcal specimens is diagnostically important and can be readily accomplished by a number of genotypic methods [63]. Multiple locus sequence typing (MLST) compares DNA banding patterns of five to seven housekeeping genes that are obtained via nuclease digest followed by electrophoresis [64,65]. Sequencing of a single gene locus has also proven to accurately identify *Staphylococcus* isolates at the species and subspecies level [66,67]. Despite the limited capacity of sequence typing of the β subunit bacterial RNA polymerase (*rpoB*) to differentiate some enteric bacteria, it is an efficient tool for species identification in coagulase-negative staph [66,67], and the genetic locus encoding superoxide dismutase (*sodA*) is reliably discriminative to the subspecies level, particularly in CoNS [68]. Shah and coworkers developed a sequence typing method for Hsp40 (*dnaJ*) with superior discrimination to the staphylococcal subspecies level [69]; however, utilization of the *tuf* locus that encodes EF-Tu, the elongation factor for peptide chain synthesis, remains the target gene of choice for diagnostic purposes as it reliably and accurately discriminates staph species and subspecies [70,71]. Speciation of *Staphylococcus* by multiplex PCR (mPCR) of the *nuc* locus, encoding staphylococcal thermonuclease, has 100% sensitivity and specificity for the subset of staph species that possess this gene, including *S. aureus*, *S. epidermidis*, *S. haemolyticus*, *S. saprophyticus*, *S. capitis*, *S. caprae*, *S. warneri*, *S. hominis*, and *S. lugdunensis* [72].

Matrix-assisted laser desorption/ionization–time-of-flight mass spectrometry (MALDI-TOFMS) is now in routine use in larger clinical labs for microbial identification, commercialized by Bruker as the MALDI Biotyper. MALDI-TOFMS accurately identifies many Gram-positive and Gram-negative bacteria, mycobacteria, and fungi to the species level through the detection and quantification of biopolymers (larger than 800 amu) [73]. To avoid misidentification of closely related species with inherent similarities, isolation of pure cultured colonies is critical [74,75,76]. Once pure cultures are obtained, rapid and accurate analysis is accomplished within one hour and relies upon matching MALDI mass spectral fingerprints to a comprehensive database of previously identified organisms, containing at least ten spectral entries for each strain [74,75,76]. Remarkable advancements in the applications of laser desorption/ionization mass spectrometry have vaulted the field of analytical chemistry to the forefront of the biomedical industry. Hou and colleagues review the most current clinical applications and propose intensive concentration on continuous database updates and enhancements to broaden the utility of mass spectral fingerprinting [77]. 

## 3. Volatile Metabolites and Breath Analysis as Emerging Diagnostics for Staph Infections

### 3.1. Principles and Advantages of VOC-Based Diagnostics

Current diagnostic procedures for staph infections have several significant limitations that ultimately complicate effective identification and eradication. Obtaining a viable bacterial sample or bacterial genomes for in vitro identification remains a significant barrier in clinical diagnostics. For example, the Centers for Disease Control and Prevention (CDC) recommends active surveillance testing of NICU patients to monitor endemic and outbreak incidents of *S. aureus* [78], but the standard nasal swab application for sample collection and mupirocin treatment causes pain, irritation, and mucosal bleeding in this vulnerable population. Sampling lower respiratory infections is also difficult in all age groups, but especially young children. The primary specimen used for diagnosing pulmonary infections is sputum, provided through a productive cough, which requires the patient to be able to expectorate on demand. Alternatives, such as the oropharyngeal swab (throat swab) or cough swab are less sensitive and less specific for many lower respiratory infections [79,80,81,82]. Bronchoalveolar lavage (BAL) is invasive and administered under anesthesia, and therefore limited in frequency of application [83]. Similar to respiratory infections, soft tissue and skin infections, deep abscesses, osteomyelitis, endocarditis, and bacteremia all require retrieval of viable and culturable specimens [53,54,55,56]. Only bacteremia and soft tissue and skin infections, including surgical site infections, are amenable to relatively simple blood collection, site swabbing or skin punch biopsy. Even more invasive and aggressive sampling techniques are required when the focal infection resides farther from the skin or blood, with deep abscesses and bone infections necessitating surgical interventions [84,85]. Once the sample is retrieved from the site of infection, it may ultimately fail to provide a pure laboratory-cultured specimen or microbial genome for identification. 

Metabolomics-based diagnostics, whether through the analysis of blood, urine, saliva, or breath, address some of the common drawbacks of standard culture-based and molecular diagnostic approaches. In the context of infections, the metabolome reflects the interactions between host and pathogen throughout the processes of infection and resolution, which can be captured as needed and analyzed for disease diagnosis, characterization, or monitoring [86,87,88]. The volatile organic compounds (VOCs) are a subset of metabolites that are low molecular-weight carbon-containing compounds, generally less than 300 amu, that readily evaporate at room temperature [89,90]. The VOC metabolites–whether detected in breath or in other ex vivo specimens–originate from cellular infectious agents and the patient, providing information about the infection in situ. Thus, VOCs can be used as culture-independent biomarkers to identify the infection etiology based on known volatile metabolome profiles for infections, eliminating the uncertainty of successful microbial growth and the lengthy time required for generating pure cultures. Development of VOC profiles for the identification of specific pathogenic microbes, especially for application to breath-based diagnostics, is an area of intensive research [91,92,93,94], and though VOC biomarkers and breath-based diagnostics are most obviously applied to the diagnosis of lung infections, they are also applicable to non-respiratory infections [95,96,97]. Breath is one of the primary waste streams of the body (in addition to urine, feces, and sweat) and is the headspace of the blood. That is, blood transports VOC metabolic waste that originates in all tissues, organs, and systems of the body to the lungs via systemic circulation, simultaneously exchanging the VOCs from the capillaries to the alveolar space when carbon dioxide is exchanged for oxygen [98]. Thus, exhaled breath contains VOCs accumulated from the body or directly produced in the alveolar, bronchial, and oropharyngeal spaces, and depending on the exhalation and breath collection method, samples from specific portions of the respiratory tract can be collected [98]. Breath tests have high sensitivity and specificity, especially when utilizing a profile of multiple VOCs as biomarkers for infection [99]. VOCs can differentiate closely-related pathogens of the same genus and have the capacity to concomitantly reveal antibiotic sensitivity or resistance [100,101]. Because breath volatiles can be detected down to picomole concentrations [102], it may be possible to detect disease prior to occurrence of symptomology.

Another advantage of volatile biomarkers and breath-based diagnostics is that breath is an unlimited resource that can be readily and frequently captured, even under the most severe physiological state, such as endotracheal intubation and assisted breathing [103,104]. This allows clinicians to capitalize on the fact that metabolites are continually generated, consumed, or excreted through a variety of pathways, and therefore their abundance is a reflection of the current state of a disease [89]. Periodic breath sampling throughout the patient’s illness and treatment would enable the clinician to follow the progression and resolution of microbial infections and other diseases in real time [105,106], and to detect adverse reactions to treatment in advance of severe complications [107]. The recent review of breath biomarkers as diagnostic tools by Pham and Beauchamp [108] expertly addresses the current strengths and limitations of diagnostic breath analysis.

### 3.2. Tools of the Trade for VOC Analysis and Clinical Detection

Due to the complexity of human breath samples and the inherent variations between individuals, a suite of VOCs comprising a biomarker profile for individual species of microbial pathogens is more advantageous for microbial identification rather than single biomarkers [109,110,111]. Two main chemical analysis strategies are employed in the discovery and translation of VOC biomarkers from bench to bedside. The first strategy identifies chemical compounds that are diagnostic of disease using instruments that include both the separation and chemical characterization of compounds (e.g., chromatography combined with mass spectrometry). Once the biomarker VOCs are chemically identified, instruments and/or methods are developed to specifically detect and quantify the diagnostic biomarkers in the clinic or the field. The second strategy omits the identification of individual VOC biomarkers and instead detects patterns of chemical information for identification of disease (e.g., direct injection mass spectrometry, sensor arrays). The common instruments and their applications to VOC biomarker discovery are described below.

#### 3.2.1. Gas Chromatography

Analytical laboratories rely upon gas chromatography (GC) as the gold standard for separation and quantification of VOCs [112,113]. The application of mass spectrometry (MS) as a detector for the GC enhances the identification of known and unknown compounds via ionization, separation, and quantitation of intact or fragmented compounds by their mass-to-charge ratios (*m/z*) [89,114]. Thus, due to its excellent volatile compound separation and structural characterization capabilities, as well as its relatively low expense and wide availability, GC-MS is the workhorse of VOC biomarker discovery and clinical measurement [112,113,115]. 

Comprehensive two-dimensional gas chromatography (GC×GC), developed in 1991 by Liu and Phillips, was the first established system in which the chromatographed sample encountered separation on two columns of different stationary phases that were divided by a modulator that gated, refocused, and injected sample fractions from the first column onto the shorter second column [116,117]. This treatment, in which co-eluting compounds in the first dimension are separated in a second dimension of chromatography, effectively increased chemical compound resolution by a factor of 10 [118], enhancing the detection of low abundance compounds in complex VOC mixtures, such as breath. In addition to the increased chromatographic resolution, GC×GC coupled with time-of-flight mass spectrometry (TOFMS), with acquisition speeds up to 500 mass spectra per second, has the capacity to deconvolute partially co-eluting constituents, further improving analytical resolution [119]. These advantages have spurred substantial growth in the application of GC×GC to breath biomarker discovery [114]. 

For GC or GC×GC analyses of breath, the VOCs are typically captured on devices containing polymer and/or graphitic sorbents (e.g., solid-phase micro-extraction (SPME), needle traps, thermal desorption tubes), which are subsequently heated to release the VOCs into the instrument for chromatographic separation and detection [120,121,122,123]. Some advantages of this approach are the pre-concentration of the breath VOCs prior to injection, the portability of the breath samples from the site of collection to the site of analysis, and the aforementioned separation capacity of chromatographic systems [123]. However, the ultimate use of breath-based diagnostics is via point-of-need analysis, for which other analytical systems are specifically designed, and can be implemented in breath-based diagnostics either for the detection of specific biomarkers discovered via chromatographic analyses, or via the development of platform-specific breathprints that are diagnostic indications of disease.

#### 3.2.2. Direct Injection Mass Spectrometry

Direct injection mass spectrometry methods introduce samples into the mass spectrometer without any prior separation by chromatography, and therefore require little or none of the solvents or reagents typically used in separations, thus representing the greenest approaches to analytical chemistry applications [124]. Three main direct injection MS methods are used for VOC analyses: proton transfer reaction mass spectrometry (PTR-MS), selected ion flow tube mass spectrometry (SIFT-MS), and secondary electrospray ionization mass spectrometry (SESI-MS). All three methods can be used for targeted analyses to detect and quantify specific biomarker volatiles that were identified in GC-MS or GC×GC-MS studies, or they can be used in an untargeted manner, in which all volatiles are analyzed, and a diagnostic profile is constructed for each disease or infection.

Developed in 1995 and applied to the clinic in 2013 [104], proton transfer reaction mass spectrometry (PTR-MS) relies upon the ionization of water and atmospheric gasses, forming the ionization gasses H_3_O^+^, O_2_^+•^, NH_4_^+^ and NO^+^, which are directed into a drift tube where they encounter sample breath compounds. Ionization of the breath compounds occurs upon collision with the ionization gasses, and a proton is transferred [125]. Sample ions are sorted by *m/z* and detected and quantified by MS. PTR-MS requires no sample preparation, so VOCs can be directly introduced into the drift tube via a vacuum airstream and analyzed in real-time. Since this equipment requires only electricity and distilled water to operate, PTR-MS has been miniaturized to enable field studies with proficient real-time quantification and identification. An early in vitro study demonstrating the diagnostic potential for bacterial headspace volatiles showed that the PTR-MS volatile fingerprints of *S. aureus* were differentiable from *Escherichia coli*, *Klebsiella pneumoniae*, *Citrobacter*, *Pseudomonas aeruginosa*, and *Helicobacter pylori* [126]. In the field, Gierschner and colleagues used real-time VOC profiling via PTR-TOFMS to monitor VOC emissions found in stable air of 596 dairy cows from one herd. VOC emissions differed in abundance based on time of day, average milk yield and infection with paratuberculosis [127]. One main disadvantage of PTR-MS is that sample compounds must have a proton affinity higher than the reagent gas to accept a proton, or lower than the reagent gas to donate a proton, and therefore not all compounds are ionizable by PTR. 

The selected ion flow tube mass spectrometry (SIFT-MS) technique, developed by Adams and Smith, produces positive ions via microwave plasma ionization of air and water vapor [128]. A current of H_3_O^+^, O_2_^+•^, or NO^+^ precursor positive ions are selected using a quadrupole mass spectrometer and injected into a helium stream [128]. Volatile organic compounds are then introduced to the selected precursor ions in a flow tube and undergo chemical ionization within a short reaction period [128]. The ions are carried downstream by helium to the mass spectrometer that records and counts precursor and product ions for quantification [129]. Smith and Španěl specifically developed this technique for quantification of trace gases in air and breath [129], and it has been employed in a proof-of-concept study by van Oort and colleagues of pneumonia in rats [130].

SESI-MS (also previously referred to as extractive electrospray ionization mass spectrometry (EESI-MS)) has a broad detection range and sensitivity and has shown great potential for detecting and quantifying semi-volatile breath compounds [131]. The principle of operation entails charge transfer between acidic or basic electrospray nanodroplets and analyte gasses and aerosols [132,133]. This technique has been applied to the identification of volatile fingerprints and breathprints of young children with CF and mouse-model studies of *S. aureus* lung infections [100,134,135,136,137]. A research group in Switzerland has initiated a multisite project in which standardization at all levels will be employed to facilitate translational applications of SESI-MS for breathborne volatiles [138]. One premise of the study is that coupling the SESI source with a high-sensitivity, high-resolution tandem mass spectrometer (i.e., Orbitrap) will enable the disambiguation of breath compounds and, thus, the detection of altered metabolic pathways, which may clarify the mechanistic link between clinical symptoms and the observed perturbance of exhaled metabolites.

#### 3.2.3. Sensor Arrays

Another method for point-of-need analysis of VOCs is the electronic nose (E-Nose), which emerged in the late 1980s and rapidly flourished by capitalizing on the development and applications of artificial intelligence, digital micro-circuitry, electronic sensor design, software enhancements and computer systems integration [139]. The prototypical E-Nose is comprised of an array of sensors that are arranged within an electrical circuit and that differentially respond to individual VOCs. The sensors conduct analog signals into a pattern-recognition assembly, where supervised learning methods are employed to teach the pattern-recognition software a diversity of odor classifications. From this training, a knowledge database is constructed, which is used to classify novel odor samples. Therefore, unlike the direct-injection mass spectrometry methods described above, the E-Nose platform is not used for targeted analyses of volatile biomarkers discovered using GC-MS or GC×GC-MS. The E-Nose technology is best suited as a point-of-need analytical platform that is programmed and routinely calibrated based on training samples to discriminate a disease-related breathprint profile from a healthy breathprint [140].

Several pilot studies have been published on the use of E-Nose in disease diagnostics [141]. To display full transparency of their discovery process and modeling methodology for application of E-Nose technology to discriminately diagnose ventilator-associated pneumonia (VAP), Chen and colleagues fully describe their technique for building an artificial intelligence algorithm suitable for clinical practice [142]. They used an endotracheal tube to access alveolar air from 59 ventilated patients, 33 of whom were diagnosed with VAP. The VAP-positive subjects included patients with positive cultures for *S. aureus*, *K. pneumoniae*, *Stenotrophomonas maltophilia*, *Acinetobacter baumannii*, *P. aeruginosa*, *E. coli*, *Candida albicans*, *Haemophilus influenzae* and *Enterobacter cloacae complex* [142], and a wide range of co-morbidities ranging from diabetes, COPD, cardiac arrest, lung cancer, and non-pulmonary infections were represented in the VAP-positive and negative cohorts. Evaluating eight machine learning algorithms, they found that the mean accuracy of the E-Nose platform for diagnosing VAP was 0.81, with sensitivity and specificity of 0.79 and 0.83, respectively. 

### 3.3. Diagnosing Staph Infections with VOC Biomarkers

#### 3.3.1. In Vitro and Animal Model Feasibility Studies

VOC biomarkers for *S. aureus* infections have been studied at all stages of biological and chemical translational development [99,100,137,143,144,145], demonstrating feasibility for diagnosing and characterizing staph infections in clinical and field settings. Based on the published analyses of *S. aureus* VOCs, ten analytes comprise a common *S. aureus* volatile suite (Table 1) [143,145,146,147,148,149,150,151,152,153,154]. All of these metabolites are produced by a broad diversity of fungi and bacteria, including coagulase-negative staphylococci, suggesting they may be produced by universal metabolic pathways [155]. However, combining the differences in the relative abundances of these common metabolites with suites of accessory metabolites yields VOC profiles that differentiate staph from other microbial taxa. In in vitro cultures, *S. aureus* has been successfully differentiated from *Acinetobacter spp.*, *Candida spp.*, *Clostridium perfringens*, *Enterobacter spp.*, *Enterococcus spp.*, *Proteus mirabilis, Klebsiella spp., P. aeruginosa, Streptococcus spp., E. coli, Burkholderia cepacia* complex, *H. influenzae*, *H. pylori*, *Citrobacter spp*., *S. maltophilia*, *Salmonella enterica*, *Serratia marcescens*, *Moraxella catarrhalis*, *Neisseria meningitidis*, *S. epidermidis*, and *S. lugdunensis* based on their volatile profiles measured using GC, direct injection MS, and sensor array technologies (Table 2) [126,142,143,144,145,146,151,153,154,156,157,158,159,160,161,162,163]. The unique and shared VOC profiles of each taxa form the foundation of in vitro detection and identification technologies being developed for clinical use (see Section 3.3.2). Differences in the volatile profiles have been extended to in vitro models of skin wound infection biofilms, where it has been shown that *S. aureus* can be differentiated from Gram-negative bacteria, such as *P. aeruginosa*, and also Gram-positive pathogens such as *Streptococcus pyogenes* [164,165,166]. *S. aureus* and *S. epidermidis* also have unique volatile profiles in vitro under a variety of growth conditions, indicating that infections caused by the former will be differentiable from non-infectious colonization by the latter [144,145,151,167,168]. 

While only a subset (25–34%) of in vitro VOCs reliably translate to in vivo detection [100], animal model studies have shown that breath VOCs can be used to identify infection etiology, even down to the strain level for the bacterial pathogen. Zhou and colleagues developed an in vivo rabbit pneumonia model and an ex vivo human lung paracancerous model to differentiate lung infections caused by *E. coli*, *P. aeruginosa*, and *S. aureus* [169]. They observed that within six hours post-inoculation of the lung tissue cultures, significant differences were detected in the VOCs produced by each of the three infection etiologies and the uninfected controls when analyzed by SPME-GC-MS. The breath volatiles from the rabbit infection models also showed significant differences when analyzed at 24 h post-inoculation, with six discriminatory VOCs translating from the in vitro to in vivo models. Mouse model studies by Zhu, Hill, and colleagues determined that SESI-MS breathprinting distinguished between seven of the most common causes of human bacterial lung infections: *S. aureus, H. influenzae, K. pneumoniae, Legionella pneumophila, M. catarrhalis, P. aeruginosa,* and *S. pneumoniae* [136]. They also demonstrated that breath VOCs could discriminate between infections caused by *P. aeruginosa* strains PAO1 vs. FRD1, and *S. aureus* RN450 [100] and that the etiology of bacterial lung infections can be correctly classified from early infection to clearance (from 6–120 h post-infection) [135]. In studies that exposed mice to live *S. aureus* and *P. aeruginosa*, non-infectious but immunogenic lysates of the bacteria, or saline controls, they found that breathprints of infections are the combination of bacterial metabolites, host metabolites that are correlated to immune response, and novel biomarkers that are created by the feedback between pathogen and host during active infection [137]. The involvement of the host immune system in generating VOC biomarkers of staph infections lends further support for the feasibility of differentiating between infections vs. asymptomatic colonization in humans and animals. 

VOC biomarkers are also being developed to identify clinically important staph strains, such as MRSA and toxigenic isolates. In 2010, Jia and colleagues performed a proof-of-concept study of methicillin-sensitive *S. aureus* ATCC 29213 (MSSA) and methicillin-resistant *S. aureus* NRS 382 (MRSA) cultivated in vitro and analyzed via SPME-GC-MS [148], concluding that VOC analysis by GC-MS was suitable for differentiating MRSA and MSSA, and that it may form the basis for an innovative and non-invasive diagnostic platform. These initial findings were strengthened by a SESI-MS/MS analysis of VOCs produced by isogenic MRSA and MSSA *S. aureus* strains-RN450 and 450M, respectively–that genetically differ only by the presence/absence of the SCC*mec* genes that confer methicillin resistance [170]. In this study Li and colleagues evaluated the in vitro bacterial metabolic perturbations caused by antibiotic treatment with ampicillin and showed that the MRSA and MSSA strains exhibited discriminately different metabolic profiles under the same growth conditions both before and after exposure to antibiotics. Further, Bean and colleagues showed that the volatilome differences between *S. aureus* RN450 and 450M are also detectable in the breathprints of mouse lung infection models caused by these strains, even without antibiotic exposure [134]. Combined, these studies suggest that VOCs may be used to both detect MRSA infections in situ prior to antibiotic treatment failure, and to subsequently monitor antibiotic treatment efficacy. VOCs have also shown promise for the detection and differentiation of enterotoxic and non-enterotoxic *S. aureus* strains [152] –an important issue for food safety–broadening the potential utility of VOC-based diagnostics for staph.

#### 3.3.2. Diagnosing Human Infections

VOC signatures detected in human biospecimens can differentiate infected vs. non-infected individuals in conditions where *S. aureus* is a prevalent etiology, with new diagnostics for VAP being a common target for volatile biomarkers. An investigation by Schnabel and colleagues of 100 patients with a clinical suspicion of VAP sampled exhaled breath from the expiratory limb of the ventilators and analyzed the VOCs using GC-TOFMS [171]. BAL diagnostic criteria confirmed VAP in 32 patients and ruled out VAP in 68. Subsequent multivariate statistical analysis of the breath VOC profiles enabled the identification of 12 compounds that discriminate VAP+ and VAP- patients with sensitivity and specificity of approximately 76% and 73%, respectively [171]. The BreathDx Consortium recently published results from a study of 93 breath samples from ventilated patients who were enrolled upon clinical suspicion of VAP [172]. They identified a panel of 10 VOCs that had a 96% negative predictive value for differentiating subjects with VAP (diagnosed via positive BAL cultures) versus those who are culture negative, with potentially important implications for reducing the over prescription of antibiotics in ventilated patients. Staph-specific biomarkers for VAP are also under development. In a pilot study of 22 mechanically ventilated patients diagnosed with VAP, 17 of which were confirmed by positive cultures, Filipiak and colleagues found important overlaps between the in vitro VOCs produced by *S. aureus*, *E. coli*, *Candida* spp. and hemolytic *Streptococcus* and the VOCs detected in patients infected by those pathogens [173]. As observed in mouse model studies, they found that roughly a third of *S. aureus* VOCs they had previously detected in vitro were found in the breath of *S. aureus*-infected patients. The ventilated patients were sampled longitudinally over three to eight days, and several patients transitioned between infected and uninfected states during the analysis. Promisingly, several of the potential breath biomarkers for *S. aureus* were detected more frequently during periods of infection vs. resolution. Similar encouraging overlaps between *S. aureus* VOCs from in vitro bacterial cultures and ex vivo specimens were seen in the analysis of mucus from sinus infections [174], though neither study contained sufficient numbers of *S. aureus*-positive subjects and samples to confirm these correlations.

The most significant progress in the development of *S. aureus* VOC biomarkers has come from studies of persons with CF lung infections. In an analysis of the VOCs detected in 154 BAL fluid samples from CF patients with a variety of lung infections, Nasir et al. built models to discriminate samples from *S. aureus* vs. *P. aeruginosa* infections (n = 59), as well as models that discriminate *S. aureus* positive vs. negative samples (n = 133) [99]. The former model included 11 VOC biomarkers that had an area under the receiver operator curve (AUROC) of 0.79, and the latter model was 8 VOCs that could discriminate staph infected vs. uninfected patients with an AUROC of 0.88. Neerincx and colleagues analyzed the breath of 18 CF patients, 13 of whom had *S. aureus* infections, and identified nine VOCs that differentiate infected and uninfected CF patients with sensitivity and specificity of 1.00 and 0.80, respectively [175]. In both studies, the *S. aureus-infected* cohort included some subjects who had co-infections with other pathogens, such as *S. maltophilia*, *H. influenzae*, fungi/yeast, and nontuberculous mycobacteria, and the *S. aureus*-negative cohort included a mix of subjects who had no detected pathogens and subjects who had other infections. The predictive ability of VOCs to differentiate *S. aureus* infected versus uninfected patients in such a complex infection landscape as CF lung disease is notable.

Several studies have demonstrated the feasibility of detecting and characterizing non-respiratory infections by VOC analysis of ex vivo specimens or in vitro cultures. In a pilot study by Rogosch and colleagues, laboratory-confirmed bloodstream infections (n = 8) were detected with 100% diagnostic accuracy via E-Nose analysis of tracheal aspirates of 28 intubated preterm neonates [176]. The preclinical detection of late-onset sepsis caused by *S. aureus* and CoNS in preterm infants is possible up to three days prior to the onset of symptoms by the analysis of fecal VOCs using high-field asymmetric waveform ion mobility spectrometry [177]. Colorimetric sensor arrays (CSAs) have been developed to detect patterns of specific VOCs from in vitro cultures, enabling the direct identification of bacteria and yeast that cause bloodstream infections during blood culture enrichment [168,178]. Lim et al. showed that a blood culture cap modified to contain 73 VOC color indicators could differentiate *S. aureus*, *S. epidermidis*, and *S. lugdunensis* from 15 other bacterial taxa after 9 h of culturing with an overall sensitivity and specificity of 95.3% and 99.7%, respectively, using a CSA library based on more than a thousand blood culture analyses [168]. CSAs can also be used to perform rapid antibiotic susceptibility testing directly from blood cultures by growing aliquots of the cultures in an antibiotic array and monitoring for bacterial growth via VOC production. Kuil and colleagues analyzed the performance of the SPECIFIC REVEAL® CSA system for antibiotic susceptibility testing of 96 positive blood cultures [101]. They observed perfect agreement with the categorical results (susceptible, intermediate, or resistant) provided by bioMérieux VITEK®2 system for infections caused by Gram-negative bacteria and 91% agreement for the Gram-positives, including *S. aureus*. The errors in the susceptibility results for the Gram-positive infections were due to the misclassification of CoNS, with five *S. epidermidis* and four other CoNS showing discrepancies for oxacillin, cefoxitin, or vancomycin.

Few research studies utilizing GC-MS analysis focus on identifying *S. epidermidis* volatiles [144,145,151,179], but as a skin commensal, there has been interest in how *S. epidermidis* VOCs contribute to the attraction of mosquitoes. Verhulst and colleagues demonstrate a profile of eight *S. epidermidis* VOCs, produced in the context of human skin, comprising a suite of semiochemicals that attract *Anopheles gambiae*, a mosquito known to carry malaria [179]. These compounds include dimethyl disulfide, butyl acetate, butyl 2-methylbutanoate, 2-pentadecanone, dimethyl tetrasulfide, dimethyl pentasulfide, hexathiepane, and the inorganic compound octasulfur. With the universality of *S. epidermidis* as a human colonizer but the paucity of information on *S. epidermidis* VOCs, a lot of work remains to characterize this bacterium (including strain-to-strain variations), and to determine the similarities and differences of its volatilome compared to its aggressively pathogenic relative, *S. aureus.*

#### 3.3.3. Diagnosing Animal Infections

Several research groups have been exploring the use of VOCs to detect *S. aureus* in symptomatic and sub-clinical mastitis in cattle [180,181,182,183,184], as well as the antibiotic resistance status of the infections. By adapting the VOC detection to E-Nose and other field-deployable detection devices [127,185], livestock can be routinely monitored to reduce the transmission of unnoticed infections, and to advance antibiotic stewardship activities through empirical selection of appropriate medications for MRSA. In response to the urgent need to proactively monitor livestock for antimicrobial resistant pathogens, Yuan and colleagues propose the implementation of high-resolution visual and olfactory sensing for enhanced perception of contagious disease among dairy cattle [184]. Their goal is to engineer a novel and heterogeneous digital intelligence structure that exploits the combination of visual and olfactory data of individual animals during milking. Asymptomatically infected udders potentially exhibit elevated temperatures that can be recorded by thermal imaging cameras networked to milking robots. During milking, VOC patterns can also be detected by E-Nose. By constructing high-performance machine learning models, these artificial intelligence systems may lead the way to innovative precision livestock management. With the potential to surveil and identify early infectious disease in single animals, this technology could interrupt the commonplace asymptomatic transmission of *S. aureus* throughout the herd. This novel approach using artificial intelligence for perception in uncovering underlying diseases enhances the One Health Antimicrobial Resistance initiative goals regarding antimicrobial stewardship while diminishing economic losses due to unanticipated infectious outbreaks.

## 4. Concluding Remarks on the Present and Future of VOC-Based Diagnostics

Staphylococci are found throughout the world in diverse ecological niches, yet *S. aureus* and *S. epidermidis* preferentially inhabit the nasal vestibulum and skin, respectively, of mammals and birds. Both *S. aureus* and *S. epidermidis* are opportunistic pathogens that infrequently infect their host; however, they are also the predominant agents of healthcare and livestock-associated infections, causing significant medical and economic burdens worldwide. Traditional diagnostic measures require the acquisition of a viable biological sample of the infectious agent through productive cough, swab, blood draw, biopsy, or endoscopy. At times, enrichment cultures fail to produce pure colonies of a living specimen, leading to repeated sample collection or non-empirical implementation of antibiotic regimens. The analysis of volatile metabolites of biospecimens such as blood, tracheal aspirates, feces, and breath can provide culture-independent diagnosis of staph infections, with breath being a primary target for the non-invasive detection of systemic VOCs produced by the infectious agent, the host, and metabolic interactions between the two.

The field of VOC-based diagnostics is poised for rapid advancement toward clinical translation. As described herein, there is ample data demonstrating the feasibility of diagnosing staph infections and other bacterial, fungal, viral, and non-infectious disease etiologies using in vitro, animal model, clinical, and field pilot studies. Significant progress has been made in developing hardware for VOC collection and analysis, optimizing analytical methods for targeted and untargeted VOC measurements, standardizing processes, and determining the biological origins of VOC biomarkers, as described in detail in the second edition of Breathborne Biomarkers and the Human Volatilome, published in 2020 [186]. Recently, new VOC diagnostic technologies were deployed in clinical and veterinary settings. For example, the SPECIFIC REVEAL Rapid AST System received Breakthrough Device Designation from the US Food and Drug Administration in Aug 2022 [187,188], and FDA authorization of the InspectIR COVID-19 Breathalyzer occurred in April 2022 [189,190].

However, in order to advance the many examples of VOC diagnostics for staph infections beyond proof-of-concept and into the clinic and field, several essentials must be addressed. For VOC fingerprint-based approaches (e.g., PTR-MS, SESI-MS, E-Nose, CSAs), comprehensive libraries of infection profiles that include many strains of each infectious species, as well as non-infectious etiologies of disease, need to be created and curated. The MALDI Biotyper platform provides probable infection etiologies based on analytical proteomic fingerprints matched to a manufacturer-supplied library that can be augmented by the end user. This very approach could be implemented for VOC fingerprinting diagnostics. For diagnostics based upon discrete suites of VOC biomarkers (rather than a global VOC fingerprint), confident identification of the discriminatory VOC profile composition is paramount to facilitating biomarker translation to different platforms. However, the ultimate demonstration of translatability depends upon the validation of VOC biomarker profiles or fingerprints developed through the analysis of independent subjects via independent instruments carried out by independent investigative teams. An interesting example for *Pseudomonas aeruginosa* was published in 2021 by Kos and colleagues in which previously published VOCs were utilized in a targeted analysis of the exhaled breath of people with cystic fibrosis (CF). The goal was to measure the sensitivity and specificity of the diagnostic for detecting *P. aeruginosa* infections, with a successful outcome of four discriminative VOCs for children with CF and *P. aeruginosa* infections [191]. With the wealth of available data for diagnosing staph infections via volatile organic compounds, a similar approach to that employed by Kos and colleagues could be developed. The most significant need for continued development of VOC diagnostics for staph infections is to increase the sizes of study cohorts from dozens to many hundreds of subjects. This increase in sampling would enable the inclusion of significant clinical confounders such as noninfectious staph colonization and co-morbidities such as diabetes mellitus, cancer, vascular diseases, and others that increase staph infection susceptibility. Growing investments directed toward VOC diagnostics by governments, philanthropic agencies, and new and existing biotechnology companies are enabling studies of this magnitude, increasing the pace at which new VOC biomarker profiles and diagnostic platforms are entering the market.

## Figures and Tables

**Table 1:** The canonical VOCs of the *S. aureus* volatilome.

IUPAC Name	Common Name	Molecular Formula	MW *	CAS *	KEGG *	References
acetaldehyde	ethanal	CH_3_CHO	44	75-07-0	C00084	[143,145,148]
ethanol	ethyl alcohol	C_2_H_6_O	46	64-17-5	C00469	[143,145,148,150,154]
methanethiol	methyl mercaptan	CH_4_S	48	74-93-1	C00409	[143,152]
propan-2-one	acetone	C_3_H_6_O	58	67-64-1	C00207	[143,145]
acetic acid	acetic acid	C_2_H_4_O_2_	60	64-19-7	C00033	[143,145,151,153]
3-methylbutanal	isovaleraldehyde	C_5_H_10_O	86	590-86-3	C07329	[143,145,148]
3-hydroxybutan-2-one	acetoin	C_4_H_8_O_2_	88	513-86-0	C00466	[146,147,150,151,153]
3-methylbutan-1-ol	isoamyl alcohol	C_5_H_12_O	88	123-51-3	C07328	[143,145,146,150,151,152,153,154]
(methyldisulfanyl)methane	dimethyl disulfide	C_2_H_6_S_2_	94	624-92-0	C08371	[143,145,146,152]
3-methylbutanoic acid	isovaleric acid	C_5_H_10_O_2_	102	503-74-2	C08262	[143,145,151,152,153]

* MW: Molecular weight; CAS: Chemical Abstracts Service; KEGG: Kyoto Encyclopedia of Genes and Genomes.

**Table 2:** Analyses of the in vitro volatilomes of *S. aureus* in comparison to other pathogens.

Ref.	VOC Detection Method	*Acinetobacter spp.*	*Acinetobacter baumannii*	*Burkholderia cepacia* complex	*Candida spp.*	*Candida albicans*	*Citrobacter spp.*	*Clostridium perfringens*	*Enterobacter spp.*	*Enterobacter cloacae*	*Enterococcus spp.*	*Escherichia coli*	*Haemophilus influenzae*	*Helicobacter pylori*	*Klebsiella spp.*	*Klebsiella pneumoniae*	*Moraxella catarrhalis*	*Neisseria meningitidis*	*Proteus mirabilis*	*Pseudomonas aeruginosa*	*Staphylococcus epidermidis*	*Stenotrophomonas maltophilia*	*Streptococcus spp.*	*Streptococcus pyogenes*
[126]	PRT-MS						x					x		x	x					x				
[142]	E-Nose		x			x				x		x	x			x				x		x		
[143]	GC-MS																			x				
[144]	GC×GC-MS																				x			
[145]	GC×GC-MS																				x			
[146]	GC-MS															x			x	x				
[151]	GC-MS											x								x	x			
[153]	GC-MS											x								x	x			
[154]	GC-IMS											x								x				
[156]	SIFT-MS											x						x		x			x	
[157]	SIFT-MS			x																x		x		
[158]	GC×GC-MS	x			x				x		x	x			x				x	x				
[159]	E-Nose											x	x				x			x			x	
[160]	E-Nose							x				x								x				x
[161]	SESI-HRMS											x	x							x		x	x	
[162]	GC-MS											x				x				x				
[163]	GC-MS					x						x												
[164]	GC-MS																			x				
[165]	GC-MS																			x				x
[166]	SIFT-MS																			x				x
[167]	IMR-MS *										x										x			
[168]	CSA **		x			x			x		x	x			x				x	x	x		x	

* IMR-MS: ion molecule reaction mass spectrometry ** CSA: Colorimetric Sensor Array.

## Data Availability

No new data were created or analyzed in this study. Data sharing is not applicable to this article.
